# Supplementary material for: Toxigenic Cyanobacteria and Microcystins in a Large Northern Oligotrophic Lake Onego, Russia
Source: Toxins (Basel). 2024 Oct 25;16(11):457. doi: 10.3390/toxins16110457 (PMC11598714; doi:10.3390/toxins16110457)
Supplement: Supplementary file 1 [file toxins-16-00457-s001.zip › toxins-3244922-supplementary.pdf]

Toxigenic Cyanobacteria and Microcystins in a Large Northern Oligotrophic Lake Onego, Russia

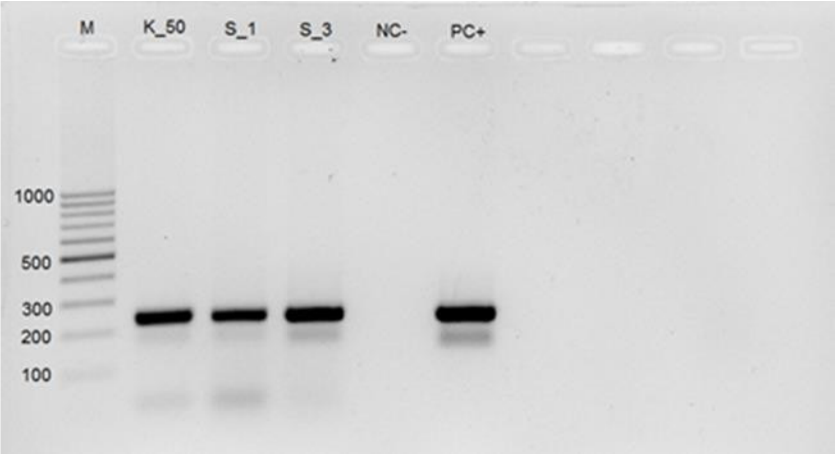

Figure S1. Amplification of the *Microcystis*-specific section of the *mcyA* gene using primers *mcyA*_MF/MR. M – DNA molecular mass marker (bp), K_50, S_1, S_3 – samples of planktonic DNA isolated from water at the studied stations of Lake Onego, NC- – negative control, PC+ – positive control.

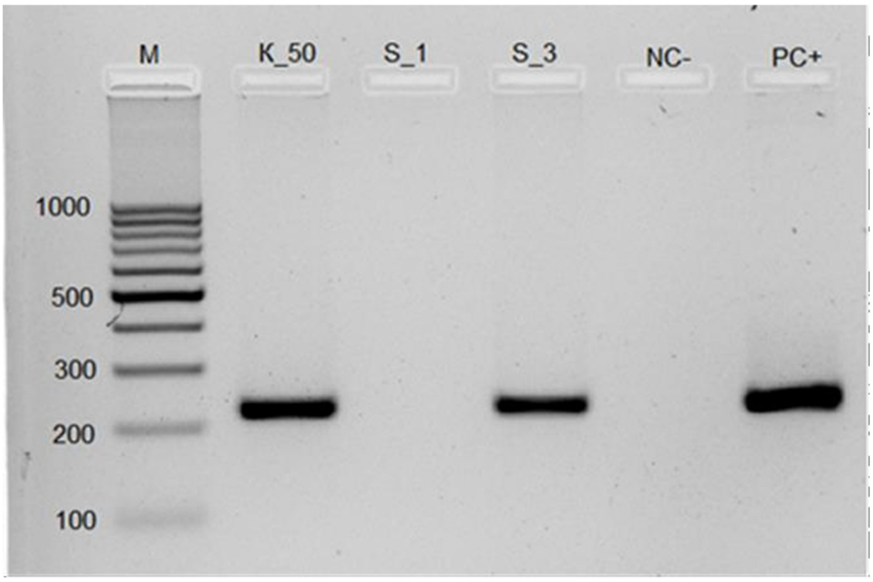

Figure S2. Amplification of the *Dolichospermum*-specific section of the *mcyA* gene using primers *mcyA*_AF/AR. M – DNA molecular mass marker (bp), K_50, S_1, S_3 – samples of planktonic DNA isolated from water at the studied stations of Lake Onego, NC- – negative control, PC+ – positive control.
